# Systematic review on peri‐operative lactate measurements to predict outcomes in patients undergoing liver resection

**DOI:** 10.1002/jhbp.727

**Published:** 2020-03-11

**Authors:** Catherine Connolly, Stefan Stättner, Thomas Niederwieser, Florian Primavesi

**Affiliations:** ^1^ Department of Visceral, Transplant and Thoracic Surgery Medical University of Innsbruck Innsbruck Austria

**Keywords:** lactate, liver resection, complications, outcome, liver failure

## Abstract

Lactate measurements have proven utility as a triage tool, therapeutic guide, and prognostic indicator, with broad use in Acute Care and transplantation. Its value in guiding therapy and predicting outcomes following liver resection is less well‐defined. This systematic review in accordance with Preferred Reporting Items for Systematic Reviews and Meta‐Analyses guidelines assessed the relationship between peri‐operative lactate levels and morbidity and mortality after liver resection. Seven relevant studies comprising 2573 patients in total were identified. Six studies assessed intra‐operative or early postoperative lactate levels, one publication examined pre‐operative levels. All studies demonstrated a significant association between peri‐operative lactate levels and adverse outcomes. The influence of pre‐operative diabetes and cirrhosis on postoperative lactate levels was shown in one study each, no study assessed the association of lactate with post‐hepatectomy liver failure according to defined criteria. The heterogeneity of study measurements and end‐points precluded a meta‐analysis from being performed. Early postoperative lactate >3‐3.7 mmol/L is associated with mortality but validation of clear cut‐off levels for outcome prediction is pending. Literature suggests lactate is a useful predictive marker for outcomes post liver surgery, especially when measured in the early postoperative phase. Further research is required to standardize the use of lactate measurements in a meaningful therapeutic manner.

## Graphical abstract

***Highlight***

In this systematic review of seven studies analyzing pre‐, intra‐ or postoperative lactate levels in 2573 patients, Connolly et al demonstrated that lactate levels at all time‐points were associated with post‐hepatectomy outcomes, and early postoperative lactate predicted mortality. Possible clinical applications include adaption of intraoperative strategies and intensification of postoperative management.

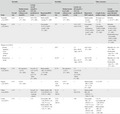

## 1 INTRODUCTION

Liver resection is the gold standard treatment for a variety of primary and secondary liver tumors.^1^, ^2^, ^3^ Technical developments combined with improvements in peri‐operative anesthetic and surgical care have led to a substantial decline in postoperative mortality over the last decades.^4^, ^5^, ^6^ However, in parallel to this trend, there are more extended resections being performed in a bid to expand curative‐intent indications.^7^, ^8^, ^9^, ^10^ As such, liver surgery is still associated with a significant rate of post‐hepatectomy liver failure (PHLF) and other severe complications.^11^, ^12^

There is a range of predictive models with general applicability and variable efficacy that can be used to select patients at a higher risk of post‐surgical complications. Some examples include the American College of Surgeons National Surgical Quality Improvement Program (ACS‐NSQIP) and the Physiological and Operative Severity Score in the enUmeration of Mortality and Morbidity (POSSUM).^13^, ^14^ Such models incorporate patient factors, procedural details, and biochemical markers. However, the complexity of these risk scores has limited their implementation into daily clinical practice. Therefore, the search for practical peri‐operative biomarkers to facilitate surgical risk stratification is of major interest.^15^, ^16^, ^17^, ^18^

Lactate measurements have demonstrated a strong correlation to clinical outcomes in critically ill patients, and are now a keystone criterion in the diagnosis of shock.^19^, ^20^ Lactate has also been established as a predictor of postoperative complications and mortality in patients undergoing cardiac surgery.^21^, ^22^ More recently, arterial lactate at the end of liver transplantation has been found to predict primary graft dysfunction,^23^ but its role following liver resection is less well‐defined. The use of lactate measurements in the setting of liver surgery warrants particular attention due to the unique role the liver plays in lactate metabolism. Pre‐operatively, lactate dynamics in patients undergoing liver surgery may be confounded by the presence of pre‐existing liver diseases.^24^ Intra‐operatively, the use of inflow‐occlusion techniques may contribute to ischemia‐reperfusion injury of the liver and subsequently impact lactate levels.^25^ Overall, the liver accounts for up to 70% of lactate clearance, and consequently liver surgery, particularly if complicated by liver failure, can have significant effects on lactate levels.^26^

The aim of this systematic review was to evaluate whether peri‐operative lactate measurement provides a simple yet accurate prediction of post‐hepatectomy outcomes.

## 2 METHODS

### 2.1 Data sources and search strategy

**Figure 1:**
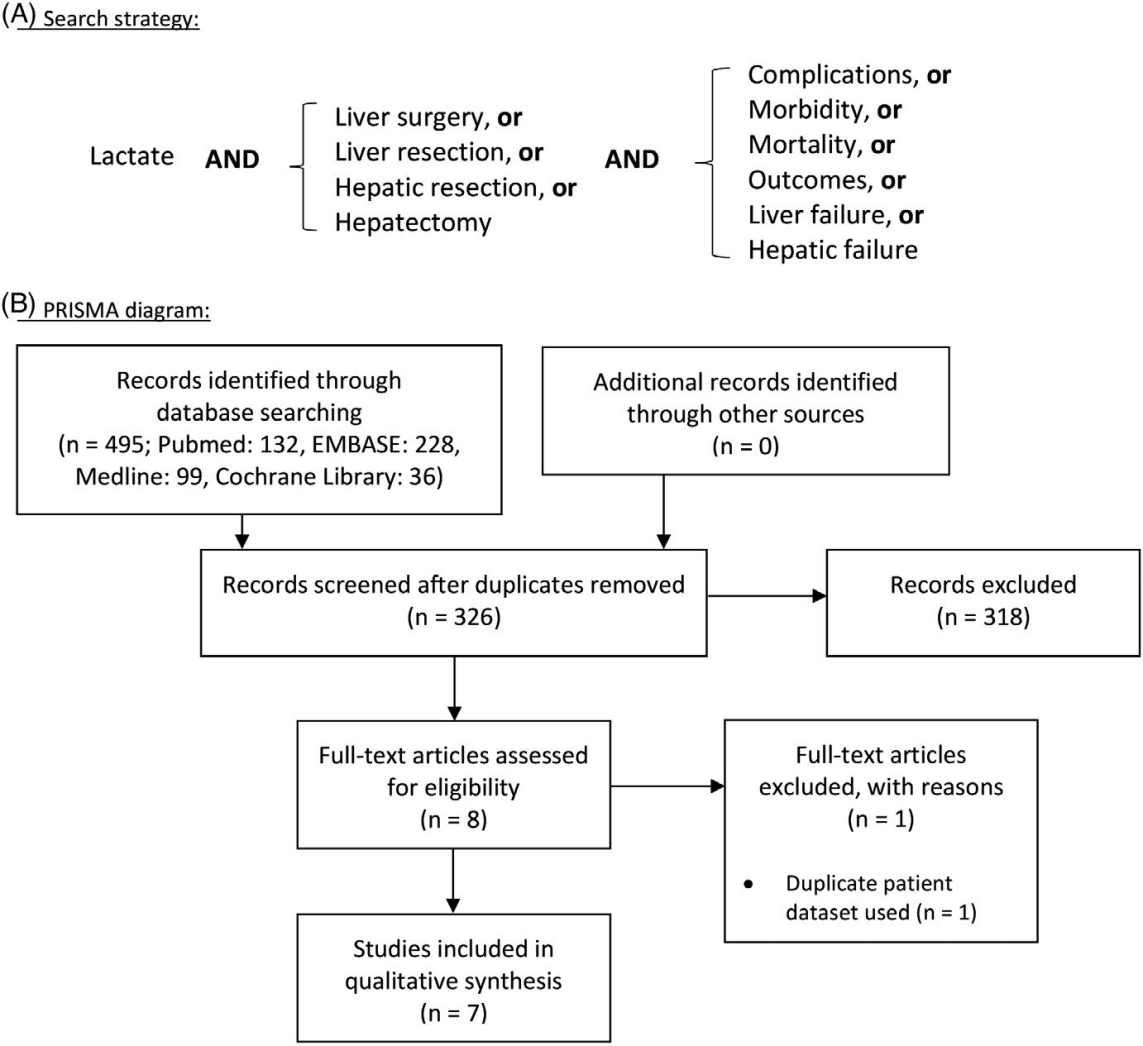
Search strategy applied for the systematic review (A) and Preferred Reporting Items for Systematic Reviews and Meta‐Analyses (PRISMA) diagram showing selection of resulting publications (B)

A systematic literature search was conducted to investigate the relationship between peri‐operative lactate levels and postoperative complications in patients undergoing liver surgery. The search was performed in accordance with the Preferred Reporting Items for Systematic Reviews and Meta‐Analyses (PRISMA) guidelines, using PubMed, EMBASE, Medline, and Cochrane databases.^27^ A search strategy was developed which included three main domains of Medical Subject Headings (MeSH) connected with Boolean operators to identify studies examining both peri‐operative lactate measurements and postoperative outcomes in patients undergoing liver surgery.^28^ The search terms used are shown in Figure 1A. Results were restricted to English‐language and human studies, and studies with a primary focus on liver transplantation were excluded. There were no restraints placed on publication status or date.

### 2.2 Data extraction and quality assessment

The search results were pooled into a citation manager, and duplicates were removed. Abstracts of the remaining studies were screened by two investigators independently to extract relevant studies. The full texts of the relevant studies were then examined, and selections were refined accordingly. The reference lists of the selected studies were also examined to identify additional publications fulfilling the inclusion criteria. Any discrepancies were resolved through discussion and subsequent consensus. The data extracted from the studies included: study design; number of patients and patient characteristics; time‐point and method of lactate measurement; operative details; and outcomes measured. All studies were graded for methodological quality using the Newcastle‐Ottawa quality assessment scale.^29^

## 3 RESULTS

### 3.1 Study selection and characteristics

The initial search strategy yielded a total of 495 records. Following application of the inclusion criteria and removal of duplicates (Figure 1B) seven studies with a total of 2573 patients (range 45–985) were included in the final systematic review (Table 1).^30^, ^31^, ^32^, ^33^, ^34^, ^35^, ^36^ With regards to the time‐point of lactate measurement, four studies measured early postoperative lactate (taken anytime from beginning of abdominal closure to within 4 hours of completion of surgery), one study measured pre‐operative lactate on Day 0, one study measured highest intra‐operative lactate, and one study measured the change between early postoperative and Day 5 levels.^30^, ^31^, ^32^, ^33^, ^34^, ^35^, ^36^

**Table 1:** Characteristics of included studies

Study	Country	Design	Sample size	Procedures	Operative diagnoses	Time of lactate measurement	Outcomes measured	Follow‐up
Watanabe et al (2007)	Japan	Retrospective cohort	151	Liver resections	—	Postoperative (Day 0)	Mortality; morbidity; length of stay; peak bilirubin	Duration of admission
Wiggans et al (2013)	United Kingdom	Retrospective cohort	488	Liver resections	Benign: 8.2% Primary malignancy: 20.7% Secondary malignancy: 71.1%	Postoperative (Day 0)	Mortality; renal dysfunction; peak bilirubin & prothrombin time; length of stay	90 d
Meguro et al (2014)	Japan	Prospective cohort	77	Liver resections	Benign: n.a. Primary malignancy: 63.6% Secondary malignancy: 36.3%	Intra‐operative (highest level)	Wound or intra‐abdominal infections	Duration of admission
Riediger et al (2014)	Germany	Prospective cohort	337	Open liver resections	Benign: 20.8% Primary malignancy: 32% Secondary malignancy: 45.7% Liver trauma: 1.5%	Pre‐operative (Day 0)	Mortality; morbidity; re‐operation	30 d
Pagano et al (2015)	Italy	Retrospective cohort	45	Extended hepatectomies	Benign: 8.9% Primary malignancy: 33.3% Secondary malignancy: 53.4% Liver trauma: 4.4%	Postoperative (Day 0 and Day 5)	Mortality; morbidity; length of stay	90 d
Vibert et al (2015)	France	Prospective cohort	519—TC 466—VC	Liver resections	Benign: 10.3% Primary malignancy: 42.2% Secondary malignancy: 45.2% Parasitosis: 2.1%	Postoperative (Day 0, 1‐4 h postoperative)	Comprehensive complication index; mortality; morbidity	90 d
Lemke et al (2017)	Canada	Retrospective cohort	490	Liver resection	Benign: 10.2% Primary malignancy: 12% Secondary malignancy: 73.4% NET: 4.3%	Postoperative (Day 0)	Mortality; morbidity; length of stay	90 d

Abbreviations: n.a., data not available; NET, neuroendocrine tumours; TC, training cohort; VC, validation cohort.

Six studies evaluated postoperative mortality and five studies assessed overall postoperative morbidity.^31^, ^32^, ^33^, ^34^, ^35^, ^36^ Three studies defined morbidity as complications falling under Clavien‐Dindo Grades III–IV, whereas the remaining four studies provided lists of their recorded complications.^30^, ^31^, ^32^, ^33^, ^34^, ^35^, ^36^, ^37^ In some of the studies, more specific outcome parameters such as re‐operation rate, length of stay, postoperative infections, renal dysfunction, and peak bilirubin were analysed.^30^, ^31^, ^32^, ^33^, ^34^, ^35^, ^36^ None of the studies specifically assessed the correlation of lactate and PHLF according to accepted definitions such as the International Study Group for Liver Surgery (ISGLS) classification or 50‐50 criteria.^38^, ^39^ The follow‐up period varied between patient cohorts—four studies reported on 90‐day postoperative outcomes, whereas one study examined 30‐day, and two studies presented in‐hospital outcomes.^30^, ^31^, ^32^, ^33^, ^34^, ^35^, ^36^

### 3.2 Methodological quality

Methodological assessment details according to the Newcastle‐Ottawa quality assessment scale are summarized in Table 2.^29^ There were nil randomized controlled trials, rather, all publications were either retrospective or prospective cohort studies with Oxford level of evidence 2b.^40^ All studies reported baseline patient characteristics and specified the enrolment timeframe. Six of the seven studies provided information regarding the indication for liver resection, intra‐operative details, and the presence or absence of pre‐existing liver disease.^31^, ^32^, ^33^, ^34^, ^35^, ^36^ One study had a notable paucity of peri‐operative details, conferring a high risk of bias.^30^ All except one study specified that arterial blood was collected for lactate measurement.^34^ None of the studies provided details on the technical aspects of lactate measurements. Three of the studies had a follow‐up period of <90 days, and all studies neglected to comment on the completeness of follow‐up or any missing data values.^30^, ^32^, ^34^ Overall, none of the selected studies addressed all features of the Newcastle‐Ottawa scale and thus an uncertain risk of bias is present.^29^, ^30^, ^31^, ^32^, ^33^, ^34^, ^35^, ^36^

**Table 2:** Methodological assessment

Study	Oxford level of evidence	Representativeness of exposed cohort	Selection of non‐exposed cohort	Ascertainment of exposure	Demonstration that outcome of interest was not present at start of study	Assessment of outcome	Follow‐up duration	Follow‐up complete
Watanabe et al (2007)	2b	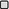	—	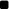	—	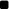	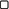	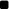
Wiggans et al (2013)	2b	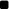	—	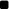	—	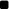	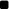	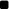
Meguro et al (2014)	2b	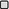	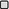	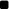	—	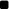	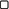	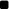
Riediger et al (2014)	2b	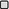	—	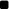	—	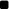	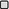	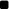
Pagano et al (2015)	2b	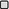	—	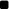	—	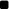	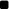	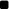
Vibert et al (2015)	2b	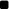	—	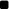	—	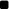	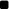	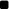
Lemke et al (2017)	2b	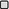	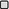	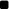	—	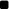	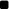	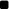

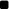
, consistent with Newcastle‐Ottawa criteria; 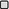, partly consistent with Newcastle‐Ottawa criteria; 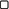, not consistent with Newcastle‐Ottawa criteria; —, not applicable.

### 3.3 Patient characteristics

The median age of the cohorts varied from 59 to 68 years. Four of the studies documented information regarding the pre‐operative status of the patients, for example in the form of their American Society of Anesthesiologists (ASA) grade or Charlson Comorbidity Index (CCI) score.^31^, ^34^, ^35^, ^36^ Pre‐operative diabetes was found to be associated with increased morbidity and mortality in three studies, with one study also finding that, on average, postoperative lactate levels were 20% higher in patients with diabetes compared to those without.^31^, ^34^, ^35^ Particular attention was also given to assessing for the presence or absence of cirrhosis in patients in six studies, either through pre‐operative investigations or an intra‐operative assessment of liver parenchyma.^31^, ^32^, ^33^, ^34^, ^35^, ^36^ The proportion of patients with cirrhosis included within these studies varied from 3.7% to 57%. While the study cohort containing 3.7% of patients with cirrhosis (n = 18) demonstrated that the condition is linked to higher post‐hepatectomy lactate levels, this relationship was not found in any of the other studies.^36^ However, cirrhosis was linked to a higher rate of postoperative complications in two other studies.^34^, ^35^ Two studies assessed the association of pre‐operative chemotherapy with postoperative lactate levels. Wiggans et al and Pagano et al showed that there was no significant difference regarding the use of any chemotherapy or application of >8 cycles with increased lactate after surgery.

### 3.4 Indications for surgery

The indications for surgery across all studies included primary and secondary liver malignancies, benign tumors, trauma, and parasitosis. One study did not specify operative pathology; however, the remaining six studies all reported liver metastases as the most prevalent diagnosis.^30^ Two studies included cases of hepatic trauma: n = 2 in the study by Pagano et al, and n = 5 in the study by Riediger et al.^33^, ^34^ Despite the possibility of additional traumatic injuries or a shocked state interacting with lactate results, nil further details were given in either study regarding the condition of the patients, or the operative procedure performed.^33^, ^34^ Nil conclusions were drawn in any of the studies regarding the impact of operative diagnosis on postoperative lactate levels.

### 3.5 Operative details

Whilst all patients underwent liver resections, one study excluded laparoscopic procedures and a further study included extended hepatectomies only.^33^, ^34^ No liver transplant donors were included in any of the studies. The total operation time was documented in five studies, and analyzed in two studies which found that longer operating times were associated with higher Day 0 postoperative lactate levels.^35^, ^36^ The use of inflow occlusion was reported in four studies and correlated with increased intra‐operative lactate levels and early postoperative lactate levels.^32^, ^36^

### 3.6 Postoperative outcomes

Table 3 shows the outcomes. Across all studies, the postoperative disposition for patients was typically the Intensive Care Unit, as lactate measurements were only available in this cohort. Mortality rates for the patients undergoing liver resections in these studies varied from 3.7% to 6.7%. Morbidity rates in the selected studies ranged from 19.2% to 48.9%. Both univariate and multivariate analyses were performed in all studies, demonstrating that lactate continued to be a predictor of outcomes after the effects of selected confounding variables were mitigated. All studies concluded with statistical significance that a single peri‐operative lactate level is a useful predictor of post‐hepatectomy outcomes.^30^, ^31^, ^32^, ^33^, ^34^, ^35^, ^36^

**Table 3:** Outcomes

Study	Mortality				Morbidity				Other outcomes	
	Overall rate	Median lactate level with/without event (mmol/L)	Lactate cut‐off (mmol/L)/AUC for prediction of event	Regression/ROC analysis	Overall rate	Median lactate level with/without event (mmol/L)	Lactate cut‐off (mmol/L)/AUC for prediction of event	Regression/ROC analysis	Length of stay: median (range)	Lactate association with: peak bilirubin/prothrombin time
Watanabe et al (2007)	6.6%	10.1/4.1 (*P* < .001)	AUC 0.86 (*P* = .008)	Multivariable: Lactate *P* = .008	45%	5.5/3.6 (*P* < .001)	—	Multivariable: Lactate *P* = .013	30 (range n.a.) ICU‐stay: *r* = .52	Bilirubin *r* = .61 (*P* < .001)
Wiggans et al (2013)	4.7%	—	—	Univariable: 0.373 ± 0.079 (coeff. ± SD; *P* < .001)	7%^a^	—	<2.0: 2.2%^a^ >6.0: 27.5%^a^	Univariable: renal dysfunction 0.324 ± 0.072 (coeff. ± SD; *P* < .001)	7 (2‐78) Univariable: 0.046 ± 0.006 (coeff. ± SD; *P* < .001)	Bilirubin: Univariable: 0.146 ± 0.017 (coeff. ± SD; *P* < .001) Prothrombin time: Univariable: 0.055 ± 0.002 (coeff. ± SD; *P* < .001)
Meguro et al (2014)										
Chronic hepatitis/Liver cirrhosis group	—	—	—	—	25%^b^	—	3.2 Sens.: 90.9% Spec.: 66.7%	AUC 0.831 (*P* < .001)	—	Bilirubin: *R* = .415 (*P* = .005) Prothrombin time: *R* = −.356 (*P* = .018)
Normal liver group	—	—	—	—	18.2%^b^	—	4.8 Sens.: 66.7% Spec.: 88.9%	AUC 0.759 (*P* = .040)	—	Bilirubin: *R* = .530 (*P* = .002) Prothrombin time: *R* = −.358 (*P* = .041)
Riediger et al (2014)	3.7%	Pre‐operative: 4.1/1.6 (*P* = .003)	Cut‐off: 4.5 >4.5: 23.5% Mort. ≤4.5: 3% Mort. (*P* = .005)	Multivariable: OR 1.47 (95%CI 1.15‐1.90) (*P* = .004)	32.7%	Pre‐operative: 2.3/1.4 (*P* < .001)	1.2 >1.2: 47.2% Morb. ≤1.2: 6.8% Morb. *P* = .001	—	—	—
Pagano et al (2015)	6.7%	8.5/4.5 (*P* = .026)	—	—	48.9% (CD III‐IV)	5.5/3.7 (*P* = .05)	—	Multivariable: OR 5.1 (95% CI: 1.1‐22) (*P* = .028)	11 (5‐107)	—
Vibert et al (2015)	4.1%	—	Cut‐off: 3.0 AUC 0.87 (training cohort)	Multivariable: OR 2.34 (95%CI 1.21‐5.39; *P* = .006)	25.1% (CD III‐IV)	—	2.8 AUC 0.76	Multivariable OR 1.96 (95%CI 1.1‐3.86; *P* = .04)		—
Lemke et al (2017)	6.3%	—	3.72	Univariable: OR 1.56 (95%CI 1.34‐1.82; *P* = n.a.) Multivariable: OR 1.52 (95%CI 1.28‐1.81; *P* < .001)	13.5% (CD III‐IV)	—	3.96	Univariable: OR 1.29 (95%CI 1.17‐1.42; *P* = n.a.) Multivariable: OR 1.19 (95%CI 1.06‐1.33; *P* < .01)	7 (6‐12) Univariable OR 1.08 (95%CI 1.05‐1.11) Multivariable: OR 1.03 (95%CI 1.00‐1.06)	—

Abbreviations: AUC, area under the curve; CD, Clavien‐Dindo; CI, confidence interval; coeff. coefficient; Morb., morbidity; Mort., mortality; n.a. not available; OR, odds ratio; ROC, receiver operating characteristics; SD, standard deviation; Sens., sensitivity; Spec., specificity.

^a^ Renal dysfunction only;

^b^ Infectious complications only.

The retrospective study published by Watanabe et al in 2007 (n = 151) demonstrated a significant difference in the mean initial postoperative lactate level between survivors and non‐survivors (4.1 mmol/L vs 10.1 mmol/L).^30^ The comparison of mean lactate levels between patients with and without postoperative complications similarly demonstrated a strong difference of 5.5 mmol/L compared with 3.6 mmol/L. The area under the receiver‐operator curves (ROC) for lactate in relationship to mortality was 0.86 and there was a significant association with severe complications such as anastomotic leakage and abdominal abscess. Whilst the relationship between lactate and PHLF was not specifically addressed in this study, multivariate linear regression analysis demonstrated that initial lactate level was a predictor of peak total bilirubin (*P* < .001).^30^

Wiggans et al^31^ examined a larger cohort of patients (n = 488) and found that early postoperative lactate was associated with all recorded outcomes: peak bilirubin, prothrombin time, length of stay, renal dysfunction, and 90‐day mortality. While the authors did not perform analyses to determine a lactate cut‐off level for predicting outcomes, they instead chose to compare two subsets of patients within their cohort—those with an initial postoperative lactate ≤2 mmol/L and those with a level >6 mmol/L. In the subset of patients with postoperative lactate >6 mmol/L, there was a significantly higher rate of pre‐operative diabetes, major resections, postoperative renal failure and mortality.^31^

In the study by Meguro et al,^32^ patients were divided into two cohorts pre‐operatively depending on whether they had evidence of chronic hepatitis/liver cirrhosis, or not. There was no difference in the highest intra‐operative lactate levels between the two cohorts, and also no significant difference in the rate of postoperative Clavien‐Dindo III–IV infectious complications.^32^, ^37^ Within the normal liver cohort, highest intra‐operative lactate was the only factor that predicted the incidence of infectious complications. In the chronic hepatitis/liver cirrhosis group, a number of variables predicted infectious complications on univariate analysis, however, highest intra‐operative lactate was the only significant predictor in multivariate analysis. In this study, lactate was associated with Pringle time, intra‐operative blood loss, operative time, postoperative prothrombin time and peak bilirubin.^32^

The study by Pagano et al^33^ was unique from the other reviewed studies as it focused on the change in lactate between postoperative Day 0 to Day 5 rather than on a single measurement. In this study, 45 patients underwent extended hepatectomies, and while the change in lactate from Day 0 to Day 5 did not predict outcomes, the initial postoperative level significantly predicted mortality and Clavien‐Dindo Grade III–IV complications.^33^, ^37^ Based on these results, the institute at which this study was performed have implemented an internal policy to limit the number of lactate measurements after major abdominal surgery.

Riediger et al^34^ enrolled 337 patients into their prospective cohort study aiming to identify pre‐operative predictors for postoperative mortality and morbidity in liver surgery. After multivariate analysis, pre‐operative elevated serum bilirubin and lactate levels emerged as predictors of adverse outcomes. The optimal pre‐operative lactate cut‐off levels for the prediction of morbidity and mortality were 4.5 and 1.2 mmol/L.^34^

In response to a systematic review by Lim et al which evidenced the poor accuracy of risk prediction models for liver resections, Vibert et al sought to build and subsequently validate a new prognostic model.^35^, ^41^ Through their trial cohort (n = 519), they calculated postoperative lactate cut‐off levels of 3.0 and 2.8 mmol/L for predicting mortality and severe morbidity respectively. Prognostic models for each end‐point were built in the training cohort using predictors identified by multivariable linear regression analysis (eg, diabetes, cirrhosis, major hepatectomy, blood loss >500 mL, etc.). These models were then applied to the validation cohort, and despite lactate levels being taken in only 60% of patients, the models demonstrated increased accuracy, sensitivity, and specificity when lactate was included.^35^

Finally, in the study by Lemke et al,^36^ early post‐hepatectomy lactate was also found to be associated with morbidity and mortality on univariate and multivariate analyses. By combining their calculated lactate cut‐off levels for morbidity and mortality, Lemke et al^36^ suggest a global level of 3.8 mmol/L for predicting adverse outcomes. In order to reduce bias, Lemke et al^36^ also examined the difference between patients who did (n = 490) and did not (n = 259) have lactate levels measured postoperatively. They found that patients who had lactate tested had a higher burden of comorbidities, lower pre‐operative hemoglobin, more extensive resections, longer operating times, and higher intra‐operative blood loss and transfusions when compared to those who did not have lactate tested.^36^

## 4 DISCUSSION

All studies in this systematic review independently demonstrated the usefulness of peri‐operative lactate measurements for predicting outcomes following liver surgery.^30^, ^31^, ^32^, ^33^, ^34^, ^35^, ^36^ Each study confirmed a statistically significant link between higher lactate levels and increased risk of postoperative mortality. While there was variability in the types of outcomes examined and length of follow‐up, all studies also demonstrated statistical significance in the correlation between lactate levels and postoperative complications.^30^, ^31^, ^32^, ^33^, ^34^, ^35^, ^36^ Notably, there were no studies found in the literature which examined the relationship between lactate levels and PHLF as defined accepted criteria.^38^ Given the unique role of the liver in lactate metabolism, combined with the prevalence and severe consequences of post‐hepatectomy liver failure, it may be pertinent to examine for a clinical correlation or pathophysiological link between them.

One study in this review examined the prognostic value of pre‐operative lactate levels on postoperative outcomes, whereas the remaining six studies measured either intra‐operative or early postoperative levels.^30^, ^31^, ^32^, ^33^, ^34^, ^35^, ^36^ In the data published by Riediger et al,^34^ there is an undefined quantity of patients who had elevated pre‐operative lactate levels which ranged up to 11.6 mmol/L. No details were given to explain the gross elevation in lactate pre‐operatively—one possible reason could be that these measurements were taken on any of the five ‘liver injury’ patients, who may have also sustained other traumatic injuries. The remaining 332 patients in this cohort had either malignant or benign tumors as the primary diagnosis warranting liver resection, and overall the pre‐operative lactate levels were found to have prognostic significance for morbidity and mortality on both univariate and multivariate analysis.^34^ However, nil other studies were identified in the literature that have examined the prognostic use of pre‐operative lactate measurements in liver surgery. Evidently, a pre‐operative measurement is not representative of intra‐operative events, and as such it is hypothesized that intra‐ or postoperative levels would have greater prognostic accuracy. Further studies are required to confirm superiority.

In the large prospective observational study by Vibert et al,^35^ lactate measurements were taken on 466 out of 777 patients (60%) undergoing liver surgery in three French centers. Similarly, in the retrospective study by Lemke et al, 490 out of 749 (65%) patients had lactate tested postoperatively. Lemke et al^36^ examined the differences between patients who did and did not have lactate measurements taken, finding that those who did have a statistically significant higher CCI, more major and longer surgeries, extra‐hepatic resections, blood loss, transfusions and a longer hospital stay. The prevalence of lactate testing following liver surgery, combined with the correlations uncovered by Lemke et al,^36^ suggests that clinicians currently apply the use of lactate testing to monitor a sub‐group of patients they perceive may have a complicated postoperative course.

Lactate has been established as useful marker in Emergency and Intensive Care settings, both as a diagnostic tool for septic shock, and as a therapeutic end‐point to guide fluid resuscitation.^42^ At present, however, there is no clear evidence base or consensus guidelines addressing acceptable lactate cut‐off levels post liver surgery. Both Lemke et al and Vibert et al were able to suggest similar lactate cut‐off levels for mortality and morbidity within their cohorts; however, the levels differ by over 1 mmol/L between studies.^35^, ^36^ Meanwhile, Riediger et al^34^ found a large disparity of 3.3 mmol/L between their calculated cut‐off levels for mortality and morbidity. In comparison, a recent study of over 12 000 patients who presented to Emergency Departments with suspected sepsis concluded that a lactate cut‐off of 2 mmol/L should be used as the threshold for initiating specific interventions and increased monitoring.^43^ It is possible that clinicians are adapting the comparatively stronger evidence base for lactate levels in other conditions such as septic shock, trauma, and other post‐surgical patients, and applying them to the cohort of post‐hepatectomy patients.

In addition to the uncertainty over what constitutes an appropriate lactate cut‐off level post‐hepatectomy, there is also the question of how clinicians respond to the perceived elevated levels. Hyperlactatemia can be contributed to via several mechanisms including tissue hypoxia, anaerobic metabolism, microcirculatory dysfunction, and reduced clearance ability.^26^ While there is an abundance of recent medical literature which emphasizes the relationship of lactate with septic shock, raised lactate levels can in fact herald a wide variety of underlying pathologies. When there is clinical evidence of inadequate tissue oxygen delivery, treatment options include volume replacement, vasopressors, and inotropes.^26^ If not, alternative diagnoses must be considered and their effects reversed or mitigated where possible. In the case of progressive liver failure, novel technologies such as the Molecular Adsorbent Recirculating System (MARS) or Prometheus machine may provide therapeutic benefit.^44^, ^45^

Effective lactate clearance has been associated with better patient outcomes across a number of studies on Emergency and Intensive Care patient cohorts.^26^, ^46^, ^47^ In this systematic review, one study examined the usefulness of continuous postoperative lactate monitoring after hepatectomy and found it to be an unreliable marker.^33^ While lactate clearance revealed no association with adverse patient outcomes in their study, the single early postoperative lactate measurement was found to predict both mortality and severe morbidity.^33^ Further studies on lactate kinetics in the post‐hepatectomy setting are required to confirm the findings of this single center cohort study.

A key limitation to this systematic review was that most studies were found to be of moderate methodological quality. In particular, many studies were inherently biased as they included only those patients who had lactate tested peri‐operatively.^30^, ^31^, ^32^, ^33^, ^34^ The application of clinical judgement in the decision to test lactate, and the subsequent exclusion from this systematic review of an unknown quantity of patients undergoing liver surgery without lactate testing clouds the analysis. Another limitation was the variability in time‐point of lactate measurements and chosen outcomes between studies, which precluded a meta‐analysis from being performed. Finally, the overall number of studies in the literature addressing this particular topic was low. It is important to broaden the evidence base on this topic, as ease of options such as venous or finger‐prick lactate testing may enable the implementation of routine postoperative lactate measurement on all post‐hepatectomy patients in the future, regardless of their disposition to the Intensive Care Unit or General Wards.^48^, ^49^

In summary, being aware of the aforementioned limitations, from a clinical perspective the results of this systematic review might be translated into daily surgical care in several different scenarios. First of all, markedly increased pre‐operative lactate levels (>4.5 mmol/L according to Riediger et al) may preclude patients from undergoing elective liver surgery, especially in case of concomitant presence of high serum bilirubin, since their expected risk for postoperative mortality exceeds 23%.^34^ In any case, careful pre‐operative anesthetic assessment is indicated in these patients. Validation of these findings in a prospective state‐of‐the‐art liver surgery cohort (excluding liver trauma cases) would be essential.

Secondly, early raised postoperative lactate values are clearly associated with increased mortality and morbidity, and they might therefore serve as a stratification tool for intensified postoperative observation on intensive care units or prophylactic measures against infections, liver failure or bleeding (antibiotics, substitution of coagulation factors, liver organ support, etc.).^30^, ^33^, ^35^, ^36^ Promising clinical trials are underway which explore the role of pharmaceutical products and bioartificial liver support systems in offering temporary support to the failing liver in the immediate postoperative phase.^50^, ^51^, ^52^, ^53^ No study has so far investigated the association of lactate cut‐offs and PHLF specifically. Regarding general outcomes, however, the median early postoperative lactate level in patients who died in the postoperative period was comparable between the studies from Watanabe et al and Pagano et al (10.1 and 8.5 mmol/L) as was the median lactate in surviving patients (4.1 and 4.5 mmol/L, respectively).^30^, ^33^ The ideal cut‐off to predict mortality as estimated by Vibert et al and Lemke et al was 3.0 and 3.72 mmol/L, respectively, and the latter group also showed that specifically patients with lactate ≥6.0 mmol/L have a dramatically increased risk of 30‐day mortality (>20%).^35^, ^36^ The results of all of these studies need to be interpreted with caution due to different patient inclusion criteria and slightly varying or ill‐defined time‐points of lactate evaluation.

Thirdly, intra‐operative measurements of lactate dynamics could theoretically facilitate adaption of surgical strategies in real‐time. For example, in complex cases requiring extensive resections plus a reconstructive phase (particularly hilar cholangiocarcinoma), an excessive increase in intra‐operative lactate values might even justify discontinuation of the procedure after parenchymal resection, temporal extracorporeal drainage of bile fluids through percutaneously placed surgical drains and final two‐stage completion with a biliary‐enteric reconstruction within 24–48 hours. Ideally, future studies designed to answer some of these questions should be performed prospectively and with international validation, include all patients undergoing minor and major liver resection, evaluate specific complications such as PHLF and collect data on various time‐points of lactate measurements to allow for analysis of lactate dynamics and determination of fixed and well‐defined time‐points for standardized use.

To conclude, while the heterogeneity of the studies meant that a meta‐analysis was not feasible, it is promising that all studies reached a positive conclusion on the usefulness of lactate for predicting outcomes post liver surgery. Further research is required to understand the strength of this correlation, the underpinning pathophysiological mechanisms, and to provide evidence‐based guidance for clinical decision‐making. There is also a need for future studies to address a particular gap in the literature pertaining to lactate and the prediction of PHLF as well as potential therapeutic intervention.

## CONFLICT OF INTEREST

All authors declare no conflict of interest for this article.

## AUTHOR CONTRIBUTIONS

All authors have contributed to design of the study, analysis of the data and drafting/revision of the manuscript, have approved the final article and are accountable in all aspects of the work.
